# An integrated multiomic single-vesicle atlas delineates graft exosome heterogeneity in chronic lung allograft dysfunction

**DOI:** 10.1038/s41392-026-02927-7

**Published:** 2026-09-17

**Authors:** Dong Tian, Xiangyun Zheng, Zengwei Yu, Dapeng Wang, Weiyang Chen, Yaling Liu, Zehui Liu, Boyang Xia, Xiaohan Jin, Haoji Yan, Ye Wu, Yongsheng Zhao, Chuanfen Zhang, Qiang Pu, Jingyu Chen, Masaaki Sato, Lunxu Liu

**Affiliations:** 1 Department of Thoracic Surgery, West China Hospital, Sichuan University, Chengdu, China; 2 Lung Transplant Research Laboratory, West China Hospital, Sichuan University, Chengdu, China; 3 Department of Intensive Medicine, The Affiliated Wuxi People’s Hospital of Nanjing Medical University, Wuxi People’s Hospital, Wuxi Medical Center, Nanjing Medical University, Wuxi, Jiangsu China; 4 Department of Cardiothoracic Surgery, The First Affiliated Hospital of Chongqing Medical University, Chongqing, China; 5 Department of Thoracic Surgery, The University of Tokyo Graduate School of Medicine, The University of Tokyo, Tokyo, Japan; 6 Department of General Thoracic Surgery, Juntendo University School of Medicine, Tokyo, Japan; 7 Institute of Thoracic Oncology, West China Hospital, Sichuan University, Chengdu, China; 8 Wuxi Lung Transplantation Centre, Wuxi People’s Hospital Affiliated with Nanjing Medical University, Wuxi, China

**Keywords:** Respiratory tract diseases, Transplant immunology

## Abstract

The underlying mechanisms of exosomes in chronic lung allograft dysfunction (CLAD) remain poorly understood. The primary challenge lies in the substantial functional heterogeneity of exosomes in CLAD. In this study, lung transplantation was initially performed, followed by the generation of a chronic rejection (CR) model in rats to simulate CLAD in humans. Exosomes from lung graft tissues were extracted, purified, and subjected to proximity barcoding assay-based single-vesicle membrane proteomic profiling. Additionally, bulk proteomic and metabolomic profiling of the graft tissues was conducted. Exosomes were categorized into 14 clusters. Compared with the syngeneic group, the CR group had significantly lower levels of clusters 7 (Nphs1^high^), 13 (Aqp1^high^), and 14 (Csf3r^high^), and higher levels of clusters 2 (Lrp2^high^ Mrc1^high^) and 4 (Nt5e^high^). The abundance of cluster 12 (Ilk^high^) exosomes was strongly correlated with pleural thickness (r = −0.90, *P* < 0.001). Cluster 12 exosomes exhibited the greatest number of related differentially expressed proteins and metabolites. The proteins correlated with cluster 12 were enriched in the “complement and coagulation cascades”, whereas the metabolites were enriched in the “glycerophospholipid metabolism”. Further verification in patients with restrictive allograft syndrome revealed the same decrease in abundance of cluster 12 exosomes as that observed in the rat model. Finally, the core molecular network of cluster 12 that contributes to pleural thickening in CLAD was constructed. In this study, the heterogeneous composition of exosomes in lung grafts was mapped to expand the understanding of their effects and to drive their application in CLAD.

## Introduction

Over the past decade, lung transplantation has seen a remarkable global increase, with more than 4000 procedures performed annually^1^. However, this growing surgical momentum is limited by the persistently bleak long-term survival of recipients. The high mortality induced by various postoperative complications limits median posttransplant survival after LTx to less than 6 years^1,2^. Chronic lung allograft dysfunction (CLAD) is the most predominant complication limiting long-term survival^3^. Nevertheless, owing to the pathophysiological particularity of the lung graft, treatment strategies for other types of transplant dysfunction are not applicable, resulting in a lack of CLAD treatment approaches^3^. Despite extensive investigation over the past decades, the pathogenesis of CLAD is still far from understood. Even with aggressive immunosuppressive therapies, the clinical outcomes remain unsatisfactory, and these regimens impose a significant risk of infections, underscoring the urgent need for clarifying the mechanism of CLAD.

CLAD is defined as an irreversible decline in lung function with no other identifiable cause, which encompasses two primary phenotypes: bronchiolitis obliterans syndrome (BOS) and restrictive allograft syndrome (RAS)^4,5^. BOS is defined by an obstructive ventilatory defect and is commonly linked to obliterative bronchiolitis (OB), which represents a type of airway fibrosis featuring intraluminal narrowing; RAS is typified by restrictive functional changes attributable to pleuroparenchymal fibroelastosis, wherein confluent areas of hypocellular collagen deposition are observed, together with the preservation and thickening of the alveolar septal elastic framework, and it is associated with pleural fibrosis^4,5^. The pathophysiology of CLAD involves complex interactions between alloimmune responses and airway remodeling processes, and chronic rejection (CR) is considered one of the most important pathophysiological processes in CLAD^6^. After being subjected to ischemia‒reperfusion and acute rejection, the MHC-mismatched rat CR model (Brown Norway to Lewis) exhibits classical chronic rejection, making it a valuable tool for understanding the underlying mechanisms and testing potential therapeutic interventions in CLAD. More importantly, this CR model exhibits classical pathological hallmarks of CLAD at 4 weeks post-transplantation and has been used for a series of CLAD studies^7,8^. Notably. on the basis of the decreased lung tissue volume, parenchymal fibrosis, and the widespread infiltration of immunocytes^8^, the model we used resembled the RAS phenotype in the clinic.

Extracellular vesicles (EVs) are defined as a collective term for lipid bilayer-enclosed particles secreted by various cells^9^. Exosomes are small EVs (50–150 nm) of endosomal origin that are released by the exocytosis of multivesicular bodies and amphisomes and are present in the greatest proportion of EVs in biological tissues^10^. The main interest in exosomes is now focused on their ability to remove molecules from cells and exchange various components between cells, which enables the involvement of exosomes in the maintenance of normal cellular homeostatic processes and the development of numerous diseases^10^. With respect to the vital pathogenic process of CLAD, exosomes play essential roles in allogeneic and autologous pulmonary antigen recognition^11–14^, mobilization and activation of innate and adaptive immune responses^15^, endoplasmic reticulum stress^16^, and epithelial-mesenchymal transition^17^ in lung grafts. In contrast, exosomes such as those derived from mesenchymal stromal cells have been shown to enhance the reconditioning of lung grafts and attenuate ischemia‒reperfusion injury^18^.

Although many vital effects of exosomes in CLAD have been reported, the underlying mechanisms remain unclear. The main difficulty in exploring the underlying mechanisms is the substantial functional heterogeneity of exosomes in CLAD because of the high diversity of components in the exosomes secreted from cells of different types and statuses^10^. The heterogeneity of exosomes is co-determined by cellular heterogeneity, the physiological status of parent cells, and the biogenesis pathways of exosomes, rendering it even more complex than cellular heterogeneity itself. Proximity barcoding assay (PBA)-based single-vesicle membrane proteomics refers to the application of the PBA—a high-throughput, multiplexed immunoassay approach—to profile extracellular vesicle membrane proteins at single-vesicle resolution^19^. Using this novel proteomics method, 260 proteins in the membranes on individual exosomes can be profiled by oligonucleotide-labeled antibodies simultaneously^19^. Briefly, each antibody is conjugated with oligonucleotides consisting of a unique protein tag to mark protein type and a molecular tag to label duplicate detection. The exosomes are captured on a surface with cholera toxin subunit B coating. The antibody-labeled oligonucleotides located on the same vesicle are assigned the same unique vesicle tag. The DNA sequences comprising barcoding information of “EV tag‒protein tag‒molecule tag” are prepared into a sequencing library for single-vesicle proteomics. Thus, in this study, we aimed to map the diverse subgroups of exosomes from rat CR grafts by using PBA-based single-vesicle membrane proteomics, and to elucidate the detailed biological effects and mechanisms underlying the effects of distinct exosome subgroups on CLAD development through multiomic analysis of derived graft tissues. The aim of this study was to effectively expand our understanding of the effects of exosomes on CLAD and promote their application.

## Results

### Histopathological evaluation of the established CR rat model

**Fig. 1:**
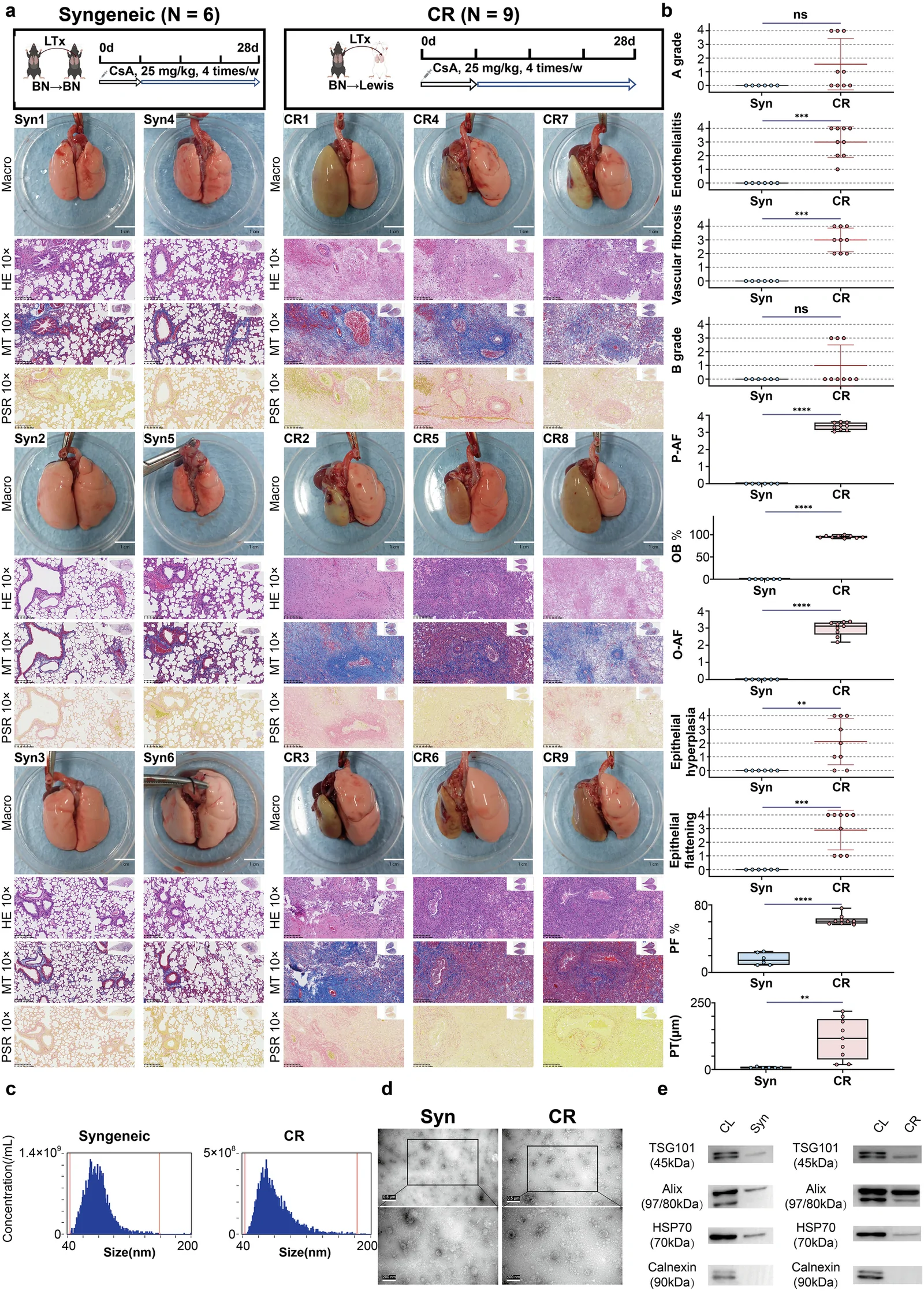
Establishment of the CR rat model and quantification and identification of graft-derived exosomes. **a** Schematic flowchart showing the establishment of the CR (lung transplant: BN to Lewis) and Syn (lung transplant: BN to BN) groups. The typical histological appearance illustrates the left lung of the rats in each group (HE staining and MT staining, objective lens: 10×). **b** The 11 plots illustrate each group’s semiquantitative assessment of CLAD pathological grade. The vertical axis (top to bottom) represents the A grade, endothelialitis, vascular fibrosis, B grade, P-AF, OB%, O-AF, epithelial hyperplasia, epithelial flattening, parenchymal fibrosis (PF%), and pleural thickness (μm), and the horizontal axis represents the different groups. **c** These plots illustrate the distribution of the exosome size in representative sample of two groups through nanoflow technology. The vertical axis represents the concentration of exosomes per mL, and the horizontal axis represents the size (nm). **d** Electron microscopy observation of the exosomes from the sample of each group (scale bars: 0.5 μm (top) and 200 nm (bottom)). **e** Western blot images of TSG101, Alix, HSP70, and calnexin in exosomes from the control lysate (CL) (lane 1) and Syn or CR groups (lane 2). *P, 0.05, **P, 0.01, ***P, 0.001, ****P, 0.0001. CR chronic rejection, Syn syngeneic, SMC smooth muscle cell, P-AF peri-airway fibrosis, O-AF obliterative airway fibrosis, OB obliterative bronchiolitis, PF parenchymal fibrosis

The experimental flow of the syngeneic (Syn) group (*N* = 6) and CR group (*N* = 9) establishment, as well as the macroscopic and microscopic manifestations of the lung grafts (including hematoxylin‒eosin (HE), Masson’s trichrome (MT), and Sirius red staining), are displayed in Fig. 1a. Briefly, the grafts of the rats in the Syn group exhibited an intact bronchiolar structure with a pseudostratified epithelial layer lining the bronchi and bronchioles, normal alveoli, and normal blood vessels. In the CR rat model group, the lung tissue volume was reduced, the surface was smooth, the elasticity was poor, and the whole lung was light brown. Microscopy analysis revealed damage to and swollen structures in the alveoli, alveolar sacs, alveolar tubes, alveolar septa, and bronchi in the CR group. The infiltration of immunocytes was observed around the vessels and bronchioles. When the MT and Sirius red staining results were combined, classical CLAD features, including bronchiolitis obliterans, the spread of fibrotic tissue to the bronchioles and vessels, and extensive pleural and subpleural fibrosis, were observed in the grafts. The levels of endothelialitis (*P* < 0.001), vascular fibrosis (*P* < 0.001), P-AF (*P* < 0.0001), OB% (*P* < 0.0001), O-AF (*P* < 0.001), epithelial hyperplasia (*P* < 0.01), epithelial flattening (*P* < 0.001), parenchymal fibrosis (*P* < 0.0001), and pleural thickness (*P* < 0.01) were significantly greater in the CR group than in the Syn group (Fig. 1b).

### Graft-derived exosome quantification and identification

Half of the samples in the Syn group (*N* = 3) and all the samples in the CR group (*N* = 9) were subjected to extraction of graft-derived exosomes using graft tissues. The particle concentration and size distribution of the exosomes extracted from each sample were determined using NanoFCM. Similar average particle sizes of the exosomes were detected in both the Syn group and the CR group (Fig. 1c and S1, Table S1), whereas the particle count per gram of graft was significantly greater in the Syn group (*P* < 0.0001) (Table S1). Transmission electron microscopy (TEM) revealed the typical cup-shaped morphology of the exosomes in both the Syn group and the CR group (Fig. 1d). Finally, with the cell lysate (CL) of graft tissues used as the control, the positive expression of three positive protein markers of exosomes (TSG101, Alix, and HSP70) and the negative expression of one negative protein marker of exosomes (Calnexin) in exosome samples from both the Syn and the CR groups were detected (Fig. 1e and S2). Equal amounts of exosomes from each graft sample were processed for PBA-based single-vesicle membrane proteomics. A total of 552488, 802118, and 568432 exosomes with PBA targeting 260 membrane proteins were captured in three samples from the Syn group, and a total of 737637, 825220, 865541, 1036906, 769291, 959316, 1009191, 938333, and 1692281 exosomes with PBA targeting 260 membrane proteins were respectively captured, respectively, were captured in nine samples from the CLAD group. With respect to the membrane proteins in the exosomes, both principal component analysis (PCA) and multidimensional scaling (MDS) revealed differences in expression patterns between the Syn and CR groups (Fig. S3a, b). Among them, the greatest fold change in upregulated expression was observed in Fn1 (logFC = 2.91, *P* = 0.038), whereas downregulated expression was observed in Cd9 (logFC = −2.74, *P* = 0.009) (Fig. S3c).

### Fourteen exosome membrane protein-based subpopulations in CLAD grafts

**Fig. 2:**
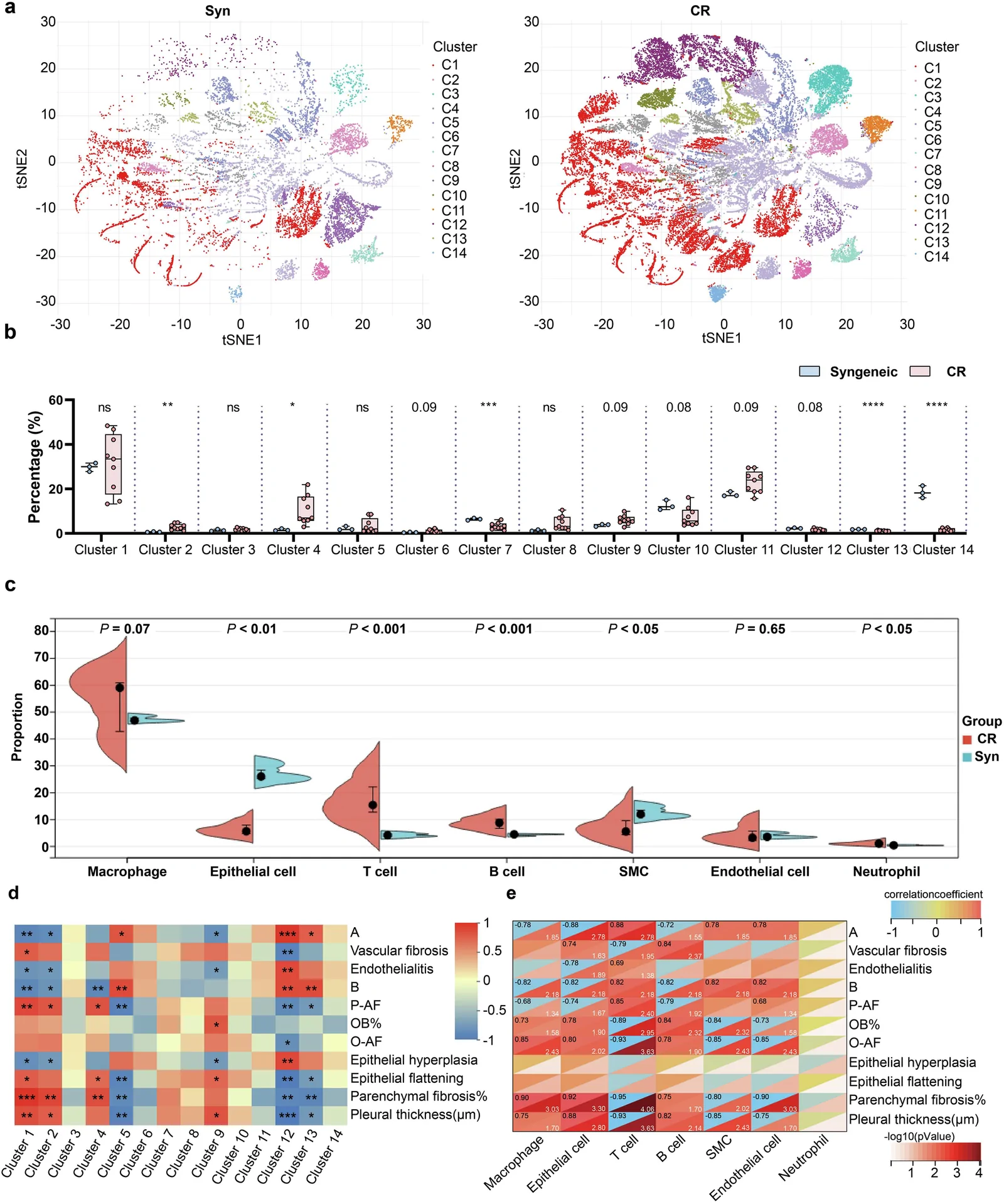
Fourteen exosome membrane protein-based subpopulations in CR grafts. **a** The t-distributed stochastic neighbor embedding (t-SNE) plot showing the heterogeneity of the exosomes from the Syn and CR groups. **b** Box plot illustrating the abundance of the 14 exosome subgroups in the Syn and CR groups. **c** Violin plot illustrating the abundance of the 8 exosome subgroups originating from different cells in the Syn and CR groups. **d** The correlation heatmap between the abundance of exosome subgroups and CR pathological grade of rat samples from the CR group. The depth of the color represents the r value (blue to red, −1 to 1). **e** The correlation heatmap between the abundance of the 8 exosome subgroups originating from different cells and CLAD pathological grade in samples from the CR group. The depth of the color (upper half of the triangle) represents the r value (blue to red, −1 to 1), and the depth of the color (lower half of the triangle) represents the *P* value. *P, 0.05, **P, 0.01, ***P, 0.001, ****P, 0.0001. CR chronic rejection, Syn syngeneic, SMC smooth muscle cell, P-AF peri-airway fibrosis, O-AF obliterative airway fibrosis, OB obliterative bronchiolitis

On the basis of the expression patterns of single-vesicle membrane proteins, the exosomes were divided into 14 clusters (Fig. 2a). The most common membrane proteins in each exosome cluster and the cell origination predictions for each cluster are displayed in Table 1. Compared with the Syn group, the CR group exhibited significantly lower abundances of cluster 7 (*P* < 0.001), 13 (*P* < 0.0001), and 14 (*P* < 0.0001), whereas the abundances of cluster 2 (*P* < 0.01) and 4 (*P* < 0.05) were significantly greater in the CR group (Fig. 2b). Compared with those in the Syn group, the abundances of exosomes from epithelial cells (*P* < 0.01) and smooth muscle cells (*P* < 0.05) in the CR group were significantly lower, whereas the abundances of exosomes from T cells (*P* < 0.001), B cells (*P* < 0.001), and neutrophils (*P* < 0.05) were significantly greater in the CR group than in the Syn group (Fig. 2c). The abundance of cluster 1 exosomes was most strongly positively correlated with the ratio of parenchymal fibrosis (r = 0.95; *P* < 0.001), whereas the abundance of cluster 12 exosomes was most strongly negatively correlated with pleural thickness (r = −0.90; *P* < 0.001) according to the correlation analysis of samples in the CR group (Fig. 2d). The abundance of exosomes from epithelial cells showed the strongest positive correlation with the ratio of parenchymal fibrosis (r = 0.92, *P* < 0.01), whereas the abundance of exosomes from T cells showed the strongest negative correlation with endothelialitis (r = −0.95, *P* < 0.01) in the correlation analysis of samples in the CR group (Fig. 2e).

**Table 1:** Cellular origin tracing of 14 exosome subpopulations

Exosome	Top 1 protein	Protein expression	Cell source	Exosome	Top 1 protein	Protein expression	Cell source
**cluster1**	Mrc1 (0.90)		Macrophage	**cluster8**	Cdh13 (1.00)		Endothelial cells
**cluster2**	Lrp2 (1.00)		Epithelial cell	**cluster9**	Cd9 (1.00)		Epithelial cell
**cluster3**	Sirpa (1.00)		T cell	**cluster10**	Cr2 (1.00)		B cell
**cluster4**	Nt5e (1.00)		B cell	**cluster11**	Havcr1 (1.00)		T cell
**cluster5**	Cd36 (1.00)		Smooth muscle cell	**cluster12**	Ilk (1.00)		T cell
**cluster6**	Mrc1 (0.61)		Macrophage	**cluster13**	Aqp1 (1.00)		Endothelial cell
**cluster7**	Nphs1 (1.00)		Epithelial cell	**cluster14**	Csf3r (1.00)		Neutrophil

### Molecular contribution of the 14 exosome subpopulations to CLAD development

**Fig. 3:**
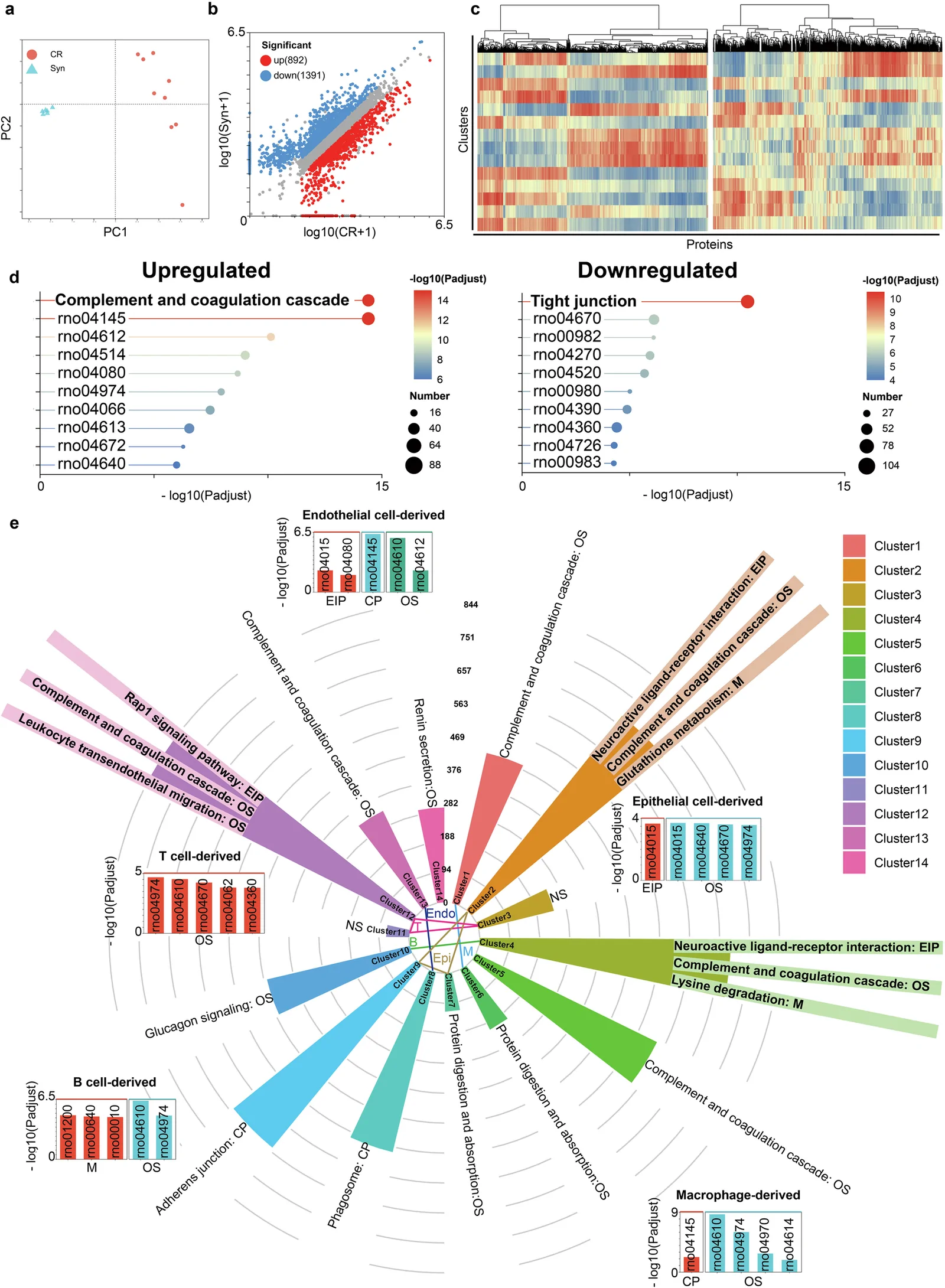
Molecular contribution of the 14 exosome subpopulations to CLAD development. **a** The PCA plot shows the differences in protein expression among the samples, and **b** the volcano plot illustrates the differentially expressed proteins in lung allografts between the Syn and CR groups. **c** The correlation heatmap between the abundance of exosome subgroups and protein expression (left, all samples; right, CR samples). The depth of the color represents the r value (blue to red, −1 to 1). **d** Lollipop plot illustrating the KEGG enrichment of upregulated (left) and downregulated (right) proteins. The depth of the color represents the -log10 *P* value (blue to red, 4 to 10). The size of the bubbles represents the number of enriched proteins. **e** The circular bar plot illustrates the number of significantly correlated proteins of 14 exosome subgroups (vertical axis) and the main KEGG enrichment results of these correlated proteins. The central lines represent the common cell sources of these clusters (blue, macrophages; brown, epithelial cells; dark blue, endothelial cells; pink, T cells; and green, B cells). The 5 columns around the circle show the KEGG enrichment for the proteins related to exosomes originating from different cells (macrophage-, endothelial cell-, T-cell-, B-cell-, and epithelial cell-derived, top to bottom). The Y-axis represents the value of -log10 *P*adjust, and the X-axis represents the pathway name. CR chronic rejection, Syn syngeneic, EIP environmental information processing, CP cellular processes, OS organismal systems, M metabolism, OPLS-DA orthogonal partial least squares discriminant analysis

Proteomic analysis of exosome-derived grafts revealed 2283 differentially expressed proteins (892 upregulated and 1391 downregulated) between the grafts in the CR group and those in the Syn group (Fig. 3a‒b). The main protein‒protein interaction network of the differentially expressed proteins was subsequently constructed (Fig. S4). The correlations between the abundance of each exosome cluster and the expression of these differentially expressed proteins in all the samples or only in the CR samples were subsequently analyzed (Fig. 3c). The “complement and coagulation cascades” pathway was most enriched among the upregulated proteins in the CR group, whereas the “tight junction” pathway was most enriched among the downregulated proteins (Fig. 3d). The related proteins of each exosome cluster were defined as the differentially expressed proteins for which r > 0.7 and *P* < 0.05. The number of related differentially expressed proteins and the most enriched KEGG pathways of each exosome cluster are displayed in Fig. 3e. The top three exosome clusters with the most related differentially expressed proteins were cluster 12, cluster 4, and cluster 2. The differentially expressed proteins related to cluster 12 were enriched mainly in the “Rap1 signaling pathway”, “complement and coagulation cascades”, “leukocyte transendothelial migration”, and “renin-angiotensin system” pathways. The differentially expressed proteins related to cluster 4 were enriched mainly in the “neuroactive ligand‒receptor interaction”, “complement and coagulation cascades”, and “lysine degradation” pathways. The differentially expressed proteins related to cluster 2 were enriched mainly in the “neuroactive ligand‒receptor interaction”, “complement and coagulation cascades”, and “glutathione metabolism” pathways. The enrichment analysis of the differentially expressed proteins related to each of the 14 clusters is displayed in Figs. S5‒7. The differentially expressed proteins related to exosomes derived from macrophages, endothelial cells, T cells, B cells, and epithelial cells were most enriched in “complement and coagulation cascades”, “phagosome”, “protein disgestion and absorption”, “complement and coagulation cascades”, and “renin‒angiotensin system”, respectively. Gene set enrichment analysis (GSEA) revealed that immune response (also enriched in proteins related to exosome clusters 1, 2, 4, 5, 8, 12, and 13) and antigen processing and presentation (also enriched in proteins related to exosome clusters 3, 8, and 12) were significantly enriched in the CR group (Fig. S8). In addition, to explore the contribution of the proteins whose expression differed the most between the two groups, the samples were identified and divided into a high-expression group and a low-expression group on the basis of Fn1 and Cd9 expression, respectively. The differentially expressed proteins between the high- and low- expression groups of Fn1 and Cd9 were enriched in “complement and coagulation cascades” (Fig. S9).

In addition, deconvolution analysis based on the proteomic data revealed significant increases in the numbers of plasma cells, activated memory CD4 T cells, follicular helper T cells, activated NK cells, M2 macrophages, resting mast cells, and neutrophils in the CR group. Conversely, decreases in the numbers of resting memory CD4 T cells, resting NK cells, monocytes, resting dendritic cells, activated mast cells, and eosinophils were observed in the CR group (Fig. S10a). Correlation analysis revealed that cluster 12 and exosomes from T cells exhibited the strongest positive correlation with activated NK cells (Fig. S10b, c). The combination of protein expression data and immunocyte-specific membrane protein marker data revealed that T-cell derived exosomes exhibited the greatest variety of differential expression among the immunocyte-derived exosomes examined (Fig. S11‒15).

### Metabolic contribution of the 14 exosome subpopulations to CLAD development

**Fig. 4:**
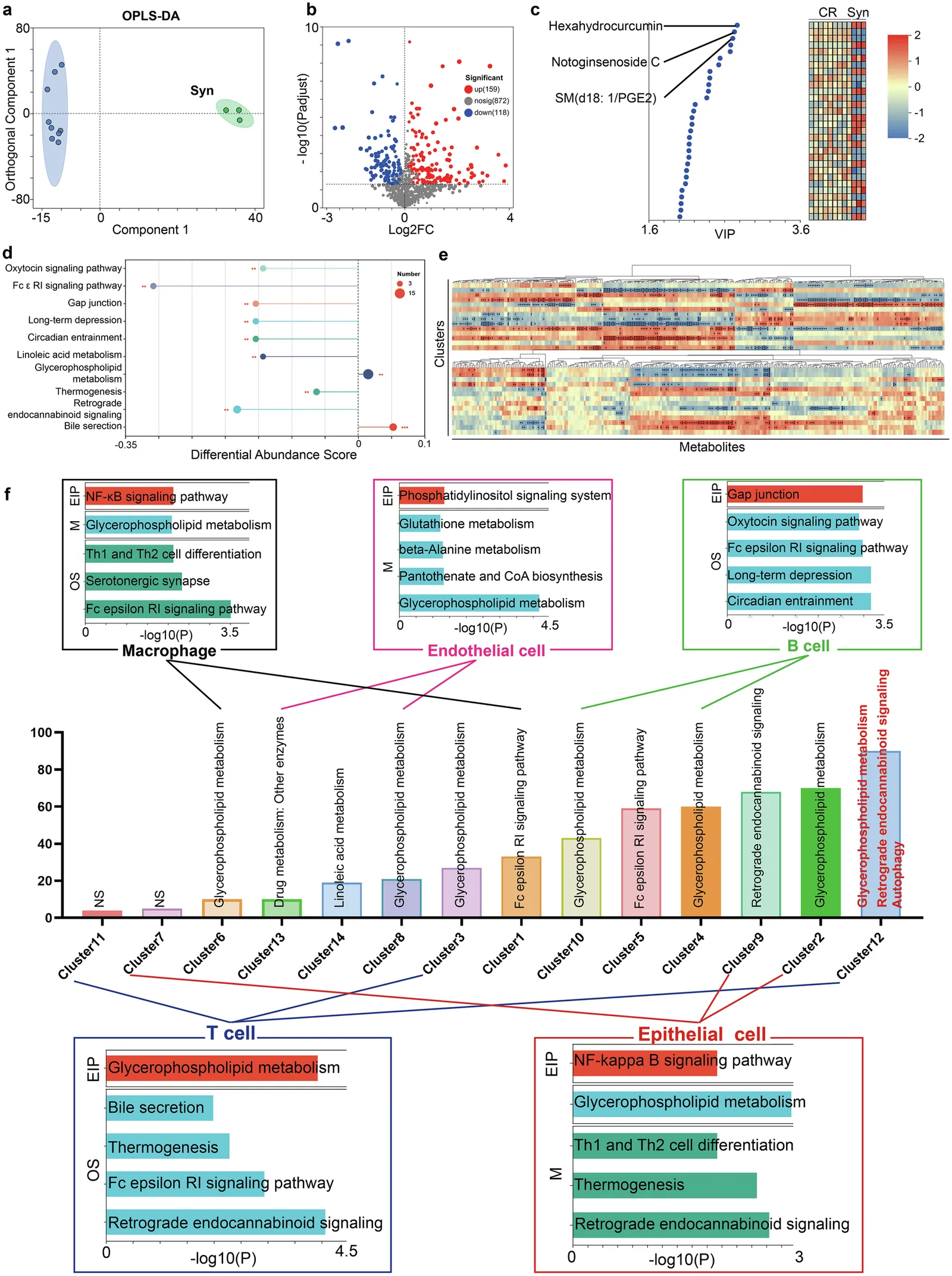
Metabolic contribution of the 14 exosome subpopulations to CLAD development. **a** PCA plot showing the differences in metabolite expression among each sample. **b** Volcano plot illustrating the differentially expressed metabolites of lung allografts between the Syn and CR groups. **c** Dot plot illustrating the VIP scores of the differentially expressed metabolites and the heatmap showing the abundance values of these metabolites. **d** Lollipop chart showing the KEGG enrichment results for the differentially expressed metabolites. The X-axis shows the differential abundance score (from −0.4 to 0.4, where a negative score represents enriched decreased metabolites). **e** All-sample (top) and solely CR-sample (bottom) correlation heatmaps between the abundance of exosome subgroups and the expression of metabolites. The depth of the color represents the r value (blue to red, −1 to 1). **f** The bar plot shows the number of significantly correlated metabolites of the 14 exosome subgroups; the text on each bar shows the top enriched KEGG pathway of the 14 exosome subgroups. The surrounding bar plots show the top 5 enriched KEGG pathways of the exosomes derived from different cells. *P, 0.05, **P, 0.01, ***P, 0.001, ****P, 0.0001. CR chronic rejection, Syn syngeneic, EIP environmental information processing, CP cellular processes, OS organismal systems, M Metabolism, OPLS-DA orthogonal partial least squares discriminant analysis

Metabolomic analysis of the exosome-derived grafts revealed 277 differentially expressed metabolites between the CR and Syn groups (Fig. 4a, b), most of which were found to be involved in lipid metabolism (glycerophospholipid metabolism) (Fig. S16a, b). Among them, hexahydrocurcumin, notoginsenoside, and SM(d18:1/PGE2) had the three highest variable importance in projection (VIP) scores (Fig. 4c). These metabolites were significantly enriched in the “Fc epsilon RI signaling pathway” (differential abundance (DA) score < 0) and “glycerophospholipid metabolism” (DA score > 0) (Fig. 4d). In the iPath analysis of the differentially expressed proteins and metabolites, lipid metabolism, energy metabolism, and carbohydrate metabolism were significantly altered (Fig. S16c). The commonly enriched pathways of the differentially expressed proteins and metabolites are displayed in Fig. S17. The **“**Fc epsilon RI signaling pathway” was enriched in both the differentially expressed proteins and the differentially abundant metabolites. Further correlation analysis between metabolites and immunocytes revealed that the expression of plazomicin was significantly positively correlated with the proportion of M2 macrophages (r = 0.98, *P* < 0.001), whereas the expression of cyclopamine was significantly negatively correlated with the proportion of gamma delta T cells (r = −0.98, *P* < 0.001) (Fig. S18).

The correlations between the abundance of each exosome cluster and the expression of these differentially expressed metabolites in all the samples or only in the CR samples were subsequently analyzed (Fig. 4e). The related metabolites of each exosome cluster were defined as the differentially expressed metabolites for which *P* < 0.05, and KEGG enrichment analysis was used to explore the metabolic contribution. The number of related differentially expressed metabolites and their enrichment results are displayed in Fig. 4f and S19. The top 3 exosome clusters with the most related differentially expressed metabolites were cluster 12, cluster 2, and cluster 9. The differentially expressed metabolites related to cluster 12 were enriched mainly in the “glycerophospholipid metabolism” and “autophagy”. Pathway map analysis revealed that cluster 12 correlated with the hub metabolites in the altered part of glycerophospholipid metabolism (Fig. S20). The differentially expressed metabolites related to cluster 2 were enriched mainly in “glycerophospholipid metabolism” and “NF‒κB signaling pathway”. The differentially expressed metabolites related to cluster 9 were enriched mainly in “retrograde endocannabinoid signaling” (Fig. 4f). The differentially expressed metabolites related to exosomes derived from T cells, epithelial cells, B cells, macrophages, and endothelial cells were most enriched in “Th1 and Th2 cell differentiation”, “NF‒κB signaling pathway”, “Fc epsilon RI signaling pathways”, “NF‒κB signaling pathway”, and “glycerophospholipid metabolism”, respectively (Fig. 4f).

### The effect of two exosome groups with opposite functions on CLAD

**Fig. 5:**
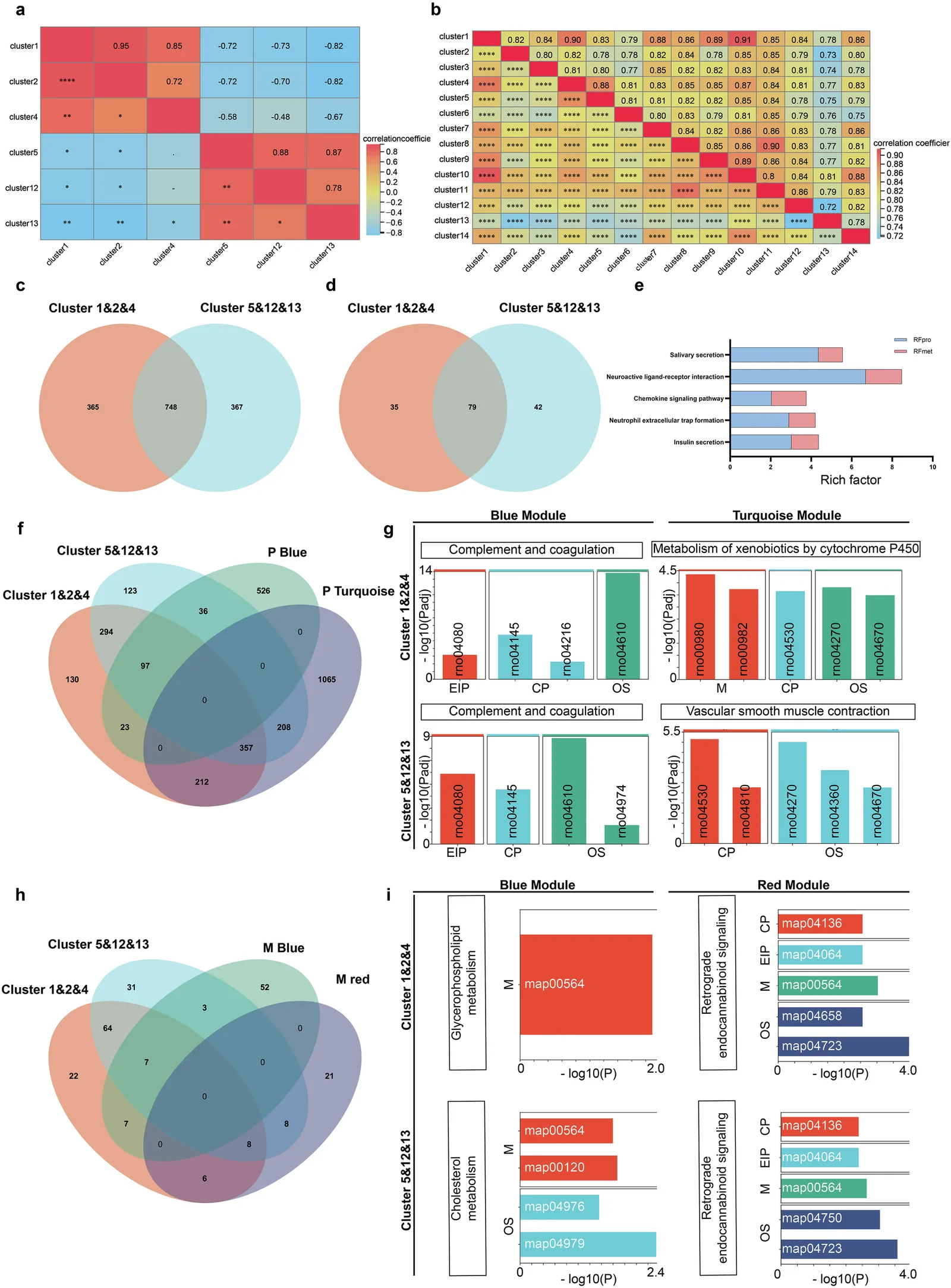
Multiomic mechanism of two exosome groups with opposite functions that affect CLAD. **a** The correlation heatmap of the abundance of clusters 1, 2, 4, 9, 5, 12, and 13 exosomes. The depth of the color represents the r value (blue to red, −1 to 1). **b** The correlation heatmap of the PBA expression patterns of the exosome subgroups. The depth of the color represents the r value (blue to red, −1 to 1). Venn diagrams of the differentially expressed proteins (**c**) and differentially abundant metabolites (**d**) related to clusters 1, 2, and 4 and clusters 5, 12, and 13. **e** The bar plots illustrate the commonly enriched KEGG pathways of the intersecting proteins and metabolites of the cluster 1, 2, and 4 group and the cluster 5, 12, and 13 group. The pink bars represent the rich factor of metabolites, whereas the blue bars represent the rich factor of proteins. **f** The Venn diagram shows the differentially expressed proteins related to the cluster 1, 2, and 4 group, the differentially expressed proteins related to the cluster 5, 12, and 13 group, the proteins in the PRO_MEblue_ module, and the proteins in PRO_turquoise_ module. **g** The bar plots illustrate the enriched KEGG pathways of the intersecting proteins of the cluster 1, 2, and 4 group, the cluster 5, 12, and 13 group, the PRO_MEblue_ module, and the PRO_turquoise_ module. The name of the top enriched pathway is labeled with text in the box. **h** Venn diagram of the differentially expressed metabolites related to the clusters 1, 2, and 4, the differentially abundant metabolites related to the clusters 5, 12, and 13, the metabolites in the PRO_MEblue_ module, and the metabolites in the PRO_turquoise_ module. **i** The bar plots illustrate the enriched KEGG pathways of the intersecting metabolites of the clusters 1, 2, and 4, the clusters 5, 12, and 13, the PRO_MEblue_ module, and the PRO_turquoise_ module. The name of the top enriched pathway is labeled with text in the box. CR chronic rejection, RF rich factor, EIP environmental information processing, CP cellular processes, OS organismal systems, M metabolism

Clusters 1, 2, 4, and 9 were positively correlated with the fibrosis pathological index and negatively correlated with the immune inflammation pathological index, whereas clusters 5, 12, and 13 exhibited the opposite correlations of those of clusters 1, 2, 4, and 9 (Fig. 2d). To further understand these two exosome groups, correlation analysis of the abundance and membrane protein expression of each cluster was performed (Fig. 5a, b). Clusters 1, 2, and 4/clusters 5, 12, and 13 were strongly associated with each other, but there were no significant correlations in abundance between cluster 9 and the other clusters (Fig. 5a). There were significant negative correlations between clusters 1, 2, and 4 and clusters 5, 12, and 13 (Fig. 5a). Further analysis based on PBA membrane protein expression revealed relatively similar expression patterns in cluster 1 and cluster 2 (Fig. 5b). To explore the antagonistic function of these two groups in CR, enrichment analysis was performed utilizing the common differentially expressed proteins and metabolites related to the cluster 1, 2, and 4 and cluster 5, 12, and 13. We found that “neuroactive ligand‒receptor interaction”, “neutrophil extracellular trap formation”, and “Rap1 signaling pathway” were enriched (Fig. 5c‒e and S21).

Multiomic functional analysis was performed to explore the contributions of these two exosome groups. Weighted correlation network analysis (WGCNA) of protein and metabolite modules revealed that PRO_MEblue_ and MET_MEblue_ exhibited the greatest positive correlations with the fibrosis index and that PRO_MEturquoise_ and MET_MEred_ exhibited the greatest negative correlations (Fig. S22‒25). Enrichment analysis was performed on the basis of the intersection of the proteins and metabolites between the differentially expressed molecules related to the two exosome groups and the molecules in the most highly correlated module. We found that the “complement and coagulation cascades” pathway was significantly enriched in the intersecting proteins of clusters 1, 2, and 4 exosome groups and PRO_MEblue_ / PRO_MEturquoise._ “Vascular smooth muscle contraction” and “leukocyte transendothelial migration” were both significantly enriched in the intersecting proteins of the cluster 5, 12, and 13 exosome group and PRO_MEblue_ / PRO_MEturquoise._ “Glycerophospholipid metabolism” and “retrograde endocannabinoid signaling” were both significantly enriched in the intersecting proteins of the clusters 1, 2, and 4 exosome group and MET_MEblue_/MET_MEred_, respectively (Fig. 5f, g). “Cholesterol metabolism” and “inflammatory mediator regulation of TRP” were both significantly enriched in the intersecting proteins of the cluster 5, 12, and 13 exosome group and MET_MEblue_/MET_MEred_, respectively (Fig. 5h, i).

### Verification and multiple mechanisms of action of Ilkhigh exosomes in the RAS

**Fig. 6:**
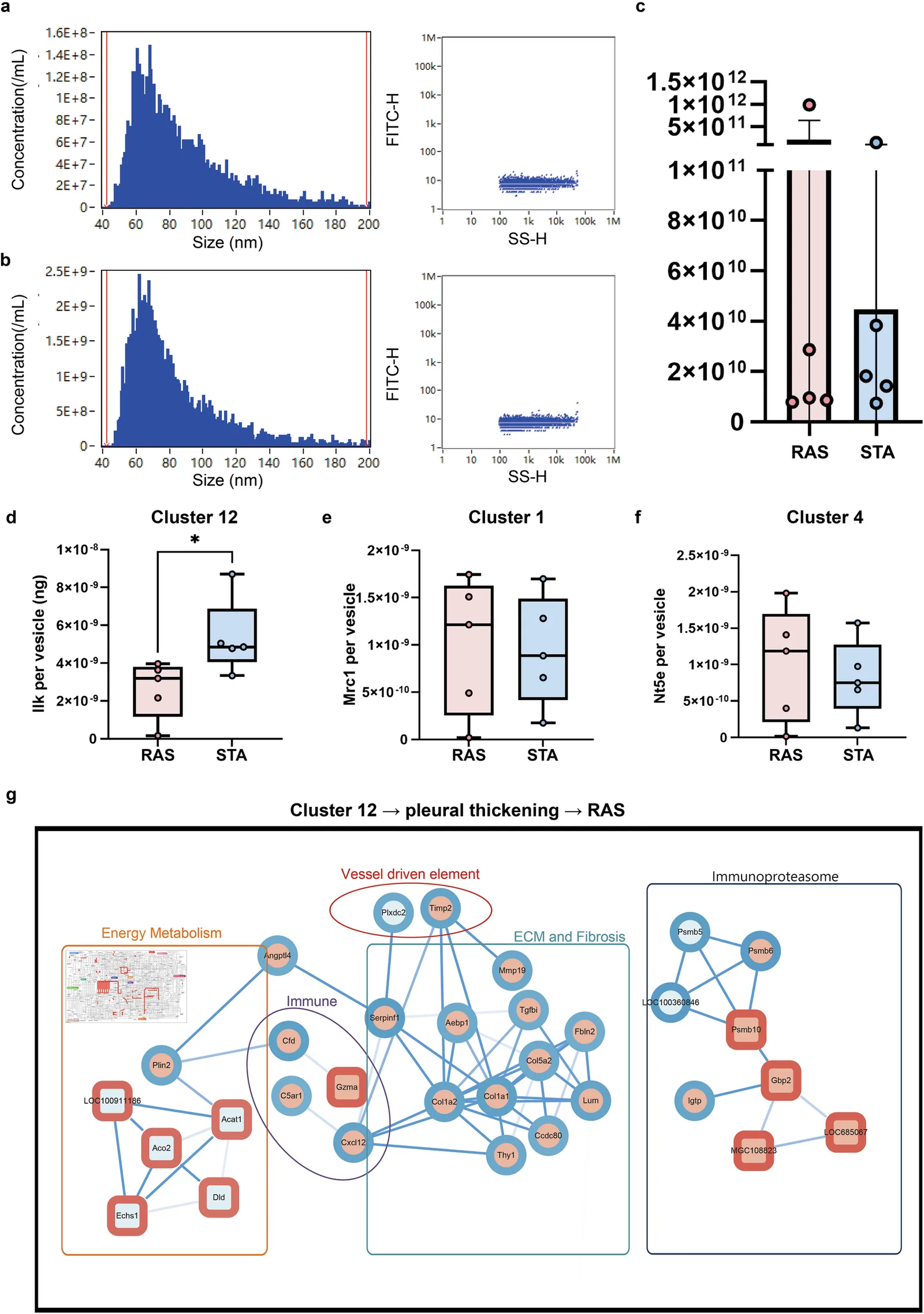
Verification and multiomic analysis of the mechanism of action of Ilk^high^ exosomes in the RAS. Nanoflow analysis of exosomes extracted from patients with RAS (**a**) and STA recipients (**b**). The left bar plot shows the distribution of exosome sizes, and the right scatter plot shows the concentration information. **c**) The bar plots illustrate the concentrations of exosomes extracted from the RAS and STA groups. Box plots showing the Ilk (**d**), Mrc1 (**e**), and Nt5e (**f**) expression per vesicle. (**g**) The hub PPI network constructed by the core correlated proteins of cluster 12 and pleural thickness. The central color of each node indicates upregulated (red) or downregulated (blue) expression in the CR group. The border color of each node indicates positive (red) and negative (blue) correlations with the abundance of cluster 12. The shape of each node indicates positive and negative correlations with the abundance of cluster 12. The iPath plots of energy metabolism illustrate the core correlated metabolites and proteins of cluster 12 and pleural thickness. RAS restrictive allograft syndrome, STA Stable patients, ECM extracellular matrix

According to both the proteomic and the metabolomic analyses, cluster 12 (Ilk^high^) exosomes were most strongly correlated with CLAD. Considering the clinical trend of RAS in the rat model we used and the strong correlation between cluster 12 and pleural thickening (an important feature of the RAS), plasma samples from 5 patients with RAS were selected to verify the expression of cluster 12 in the clinic, whereas 5 stable patients were selected as the controls (Table 2). Nanoscale flow technology revealed no significant difference in the number of extracted exosomes between the two groups (Fig. 6a‒c, Fig. S26). The membrane protein marker expression per vesicle of clusters 12 (Ilk), 1 (Mrc1), and 4 (Nt5e) was assessed by ELISA. Ilk expression per vesicle in RAS patients was significantly lower than that in stable patients (*P* < 0.05) (Fig. 6d), whereas no significant difference in Mrc1 or Nt5e protein expression per vesicle was detected (Fig. 6e, f).

**Table 2:** Baseline characteristics of the patients

		Patient			
	Overall, *N* = 10^a^	Stable *N* = 5 (50%)^a^	RAS *N* = 5 (50%)^a^	statistics	*P* value^b^
**Recipient age at transplant years**	56.60 (5.78)	58.40 (5.68)	54.80 (5.89)	0.98	0.354
**Recipient sex**					
Male	10 (100.00%)	5 (100.00%)	5 (100.00%)		
**Recipient BMI**	23.52 (1.48)	24.03 (1.95)	23.00 (0.67)	1.12	0.296
**Recipient Native lung disease**					>0.999
IPF	8 (80.00%)	4 (80.00%)	4 (80.00%)		
COPD	2 (20.00%)	1 (20.00%)	1 (20.00%)		
**Recipient Transplant type**					
Double lung transplant	10 (100.00%)	5 (100.00%)	5 (100.00%)		
**Donor/recipient CMV mismatch status**					>0.999
D+/R−	4 (40.00%)	2 (40.00%)	2 (40.00%)		
D−/R−	6 (60.00%)	3 (60.00%)	3 (60.00%)		
**Donor age**	45.00 (8.19)	46.00 (7.21)	44.00 (9.82)	0.37	0.723
**Donor sex**					
Male	10 (100.00%)	5 (100.00%)	5 (100.00%)		
**Donor ever smoker**					>0.999
Normal	7 (70.00%)	3 (60.00%)	4 (80.00%)		
Ever smoker	3 (30.00%)	2 (40.00%)	1 (20.00%)		
**Time from transplant to sampling days**	332.60 (69.91)	340.20 (66.40)	325.00 (80.28)	0.33	0.753
**Presence of respiratory symptoms**					>0.999
Acute	8 (80.00%)	4 (80.00%)	4 (80.00%)		
Chronic	2 (20.00%)	1 (20.00%)	1 (20.00%)		
**Last available FEV1**	1.95 (0.52)	2.35 (0.22)	1.54 (0.41)	3.89	**0.005**
**Median drop from CLAD baseline**	−15.00 [−28.10, −2.15]	−2.10 [−2.30, −1.90]	−28.30 [−29.10, −27.50]	25.00	**0.008**
**CMV PCR**					0.206
None detected or below quantification threshold	5 (50.00%)	4 (80.00%)	1 (20.00%)		
Positive	5 (50.00%)	1 (20.00%)	4 (80.00%)		
**Cyclosporine/tacrolimus**					>0.999
Cyclosporine	2 (20.00%)	1 (20.00%)	1 (20.00%)		
tacrolimus	8 (80.00%)	4 (80.00%)	4 (80.00%)		
**Prednisone dose mg/day**	15.00 [10.63, 40.00]	10.00 [10.00, 15.00]	40.00 [15.00, 40.00]	4.50	0.110
**Prior AR treatment within 3 months**					0.167
None	7 (70.00%)	5 (100.00%)	2 (40.00%)		
Yes	3 (30.00%)	0 (0.00%)	3 (60.00%)		
**Prior antimicrobial treatment within 3months**					
Yes	10 (100.00%)	5 (100.00%)	5 (100.00%)		

^a^Mean (SD); n (%); Median [IQR]

^b^Two Sample t test; Fisher’s exact test; Wilcoxon rank sum exact test; Wilcoxon rank sum test

*BMI* Body Mass Index, *IPF* Idiopathic Pulmonary Fibrosis, *COPD* Chronic Obstructive Pulmonary Disease, *CMV* Cytomegalovirus, *FEV1* Forced Expiratory Volume in 1 second, *CLAD* Chronic Lung Allograft Dysfunction, *PCR* Polymerase Chain Reaction, *AR* Acute Rejection

To further clarify the molecular contribution of cluster 12 to pleural thickening, we combined all the correlation data (exosomes, pathological grade, protein, immunocytes, and metabolites) to perform a multiomics analysis (Fig. S27). On the basis of the combined correlation value of molecules with cluster 12 and pleural thickening, the top 100 proteins and metabolites were identified. We mapped the core protein‒protein interaction network and iPath network of these molecules (Fig. 6g). Interestingly, the proteins that regulate energy metabolism (the core protein, Aco2), immunokines (Cfd, Gzma, C5ar1, and Cxcl12), the proteins that construct the ECM and fibrosis (the core proteins, Col1a1 and Col5a2), and two vessel-driven elements (Plxdc2 and Timp2) formed a close-knit network (Fig. 6g).

## Discussion

This is the first study to comprehensively investigate the heterogeneous composition of exosomes originating from CLAD grafts. In this study, through PBA-based single-vesicle membrane proteomics, we mapped diverse subgroups of exosomes from rat CR grafts and identified 14 novel exosome subgroups involved in CR. Pathology grading, single-vesicle membrane proteomics, bulk-proteomics, and bulk-metabolomics were subsequently combined to elucidate the biological contributions of the distinct exosome subgroups to CLAD development. Among them, clusters 1, 2, and 4 contributed mainly to fibrosis, whereas clusters 5, 12, and 13 contributed mainly to inflammation and immune attack. The altered expression in cluster 12 (Ilk^high^) exosomes was further verified in the plasma samples of patients with RAS, and the core network of cluster 12 contributing to pleural thickening was constructed.

The primary concern in this study was the variation in the abundance of the 14 exosome subgroups. Compared with the Syn group, the CR group demonstrated significantly lower abundances of clusters 7, 13, and 14. Conversely, clusters 2 and 4 exhibited higher abundances. A borderline significant difference was also observed in clusters 6, 9, 10, 11, and 12. On the basis of the enrichment analysis, the related proteins of most of these clusters were enriched in the immune response and rejection pathways. These findings suggest that alterations in the abundance of exosome subgroups have pathophysiologic implications in CLAD, which corroborates previous reports of the important role of the LKB^+^ exosome subgroup^20^ and the NK cell-derived exosome subgroup^15^. The disparity in exosome subgroup abundance can be attributed to two main factors: changes in the abundance of the originating cells of these exosomes and alterations in the rate of exosome secretion. In our cell source analysis, the increased abundance of T cell-, B‒cell-, and neutrophil-derived exosomes, as well as the decreased abundance of epithelial cell- and smooth muscle cell-derived exosomes, matched the immunocyte alteration observed in the deconvolution results in this study and earlier reports on CLAD^21,22^.

The correlations between the cluster abundance and pathological features revealed two exosome groups with opposite functions: clusters 1, 2, and 4, and clusters 5, 12, and 13. Clusters 1, 2, and 4 were positively correlated with the fibrosis pathological index and negatively correlated with the immune inflammation pathological index, whereas clusters 5, 12, and 13 exhibited the opposite correlation patterns. Further exploration of these two groups is meaningful because of the severe difficulty in the precise regulation of immune inflammation and fibrosis in CLAD therapy^23,24^. Our analysis revealed that these clusters originated from different cells, but the abundance correlation between them was strong, which might be related to the intercellular crosstalk between the source cells, as has been reported in pulmonary diseases^25^. However, few reports on CLAD exist. Notably, the related proteins and metabolites of clusters 1, 2, 4, 5, 12, and 13 were enriched in the “complement and coagulation cascades” and “glycerophospholipid metabolism” pathways. Given that the importance of the complement system and glycerophospholipid metabolism has been verified in CLAD^26,27^, these findings suggest an antagonistic effect of the two groups on the common key pathway in CLAD. Further enrichment analysis of intersecting proteins and metabolites revealed possible upstream signals, such as the Rap1 signal, which plays a role in complement receptor CR3-dependent phagocytosis^28^. Notably, as this study focused on the contribution of individual exosome subclusters to CLAD progression, correlation analyses with pathological features were performed exclusively within the CR group; expression differences between the Syn and CR groups do not necessarily imply a correlation with disease severity.

Among the 14 exosome clusters, cluster 12 displayed the greatest molecular contribution at both the protein and metabolic levels. Further clinical verification in patients with RAS suggested a decrease in the abundance of cluster 12 exosomes. Notably, cluster 12 demonstrated the most pronounced negative correlation with pleural thickening, a characteristic pathological change associated with RAS^5^. Our multiomic analyses revealed that cluster 12 is involved in the regulation of complement and coagulation cascades, leukocyte transendothelial migration, the renin-angiotensin system, and autophagy, all of which have been previously identified as significant responses in CLAD^29,30^. There was a decreasing trend in the abundance of cluster 12 in the CR group in our study, which indicates the possible protective role of cluster 12 in CLAD. However, the marker of cluster 12, Ilk, is a scaffold protein that promotes the migration of immunocytes and the transformation of various cells into myofibroblasts in allograft rejection^31^. Yue et al. reported that Ilk^+^ exosomes activate the NF‒κB pathway in endothelial cells, resulting in cardiac inflammation and injury^32^. This paradoxical effect of cluster 12 is difficult to explain on the basis of the current evidence. A possible explanation is that the Ilk^high^ exosomes play a specific role in the lung allograft, or that the decreased secretion of the Ilk^high^ exosomes could indicate the increased connectivity and transformation of Ilk^+^ cells.

A core network was established to account for the specific role of Ilk^high^ cluster 12 exosomes. For the most important module of this network, the energy metabolism (Aco2 as the core) and vessel-driven elements (Plxdc2 and Timp2) influenced the key fibrosis factors and complements in CLAD. Aco2 maintains cellular energy metabolic homeostasis through the mitochondrial tricarboxylic acid cycle^33^, which has been reported to attenuate the ischemia–reperfusion injury of lung transplantation^34^, but there are no reports on this protein in CLAD. In this study, we found the downregulated expression of Aco2 in the CR rat model, and Aco2 expression exhibited a significant positive correlation with cluster 12 abundance and a negative correlation with pleural thickness. The network indicated a possible mechanism regulated by cluster 12 in CLAD, with a decline in Aco2 shifting cellular metabolism from oxidative phosphorylation toward glycolysis and lipid synthesis^33^. In response to lipid overload, cells initiate a defensive mechanism involving Acat1 (which esterifies cholesterol)^35^ and Plin2 (which coats lipid droplets)^36^, however, this instead establishes an aberrant lipid homeostasis. Sustained metabolic stress induces the release of Angptl4, which on one hand impedes residual lipid clearance and on the other hand directly activates profibrotic signals, including Serpinf1^37^.

For the vessel-driven elements, we focused on Plxdc2, which exhibited significantly downregulated expression in CR group in our study. This finding was in accordance with a recent report by Brocard et al.^38^, whose single-cell RNA sequencing of patients with CLAD showed a downregulation of expression of Plxdc2 in fibroblasts and lung epithelium in patients with CLAD compared to healthy controls. Serpinf1 combined with Plxdc2 has been shown to promote apoptosis in endothelial cells to suppress angiogenesis^39^. However, there are no studies on the mechanism of Plxdc2 in CLAD. For our correlation analysis, Plxdc2 expression was negatively correlated with cluster 12 and positively correlated with the pleural thickness. This finding implies a possible mechanism regulated by cluster 12 in which Plxdc2 loss suppresses endothelial apoptosis in response to upregulated expression of Serpinf1 in CLAD, thereby playing a role in vessel protection to suppress pleural thickening in RAS. Further experimental reports will be needed to address this possible mechanism.

Limitations exist for this study. (i) all CR rats were sacrificed at the posttransplant week 4, so these multiomics results only showed the alterations in the most classical CLAD model, especially the RAS phenotype; (ii) the molecular contribution of each exosome cluster was based only on the correlations, and we are conducting further experiments to verify their causal roles; (iii) the experiments conducted on rats might not accurately reflect the efficacy for human applications; (iv) the immune cell analysis was derived from deconvolution of proteomics data, which, due to technical limitations, may not fully capture the exact immune cell composition within the tissue; and (v) the rat model was sacrificed at a single time point (4 weeks post-transplantation), a stage characterized predominantly by chronic rejection and established fibrosis, and could only address the immune complexities of CR during this period; therefore, we will conduct a multiperiod study in future research.

In conclusion, this study mapped the heterogeneous composition of exosomes in CR grafts, identified 14 novel exosome subgroups derived from lung allografts, and ultimately revealed the pathological and molecular contributions of each exosome subgroup to CLAD. These results expand the understanding of the effects of exosomes on CLAD and drive their application.

## Methods

### Institutional review board approval

All animal experiments were conducted using protocols approved by the Ethics Committee of West China Hospital (20221229003). All clinical studies were approved by the Ethics Committee of Wuxi People’s Hospital (KY23051).

### Rat and rat CR models

Male Lewis and Brown Norway rats were purchased from SiPeiFu Biotechnology (Beijing, China). All the rats were specific-pathogen-free inbred males weighing 250–300 g (10–12 weeks old) and were kept on a 12-hour light-dark cycle with free access to food and water. Orthotopic left LTx was performed in rats as previously described^40^. A CR rat model was established on the basis of previously published methods^8^. Briefly, LTx (Brown Norway to Brown Norway) was performed in the Syn group, whereas MHC-mismatched LTx (Brown Norway to Lewis) was performed in the CR rat model groups. After LTx, all the rats were treated with CsA four times weekly at posttransplant week 1. All the rats were sacrificed at the end of posttransplant week 4.

### Patient selection and sample extraction

The plasma samples were obtained from 10 patients who were regularly followed up and reexamined between December 2025 and February 2026. The samples were collected at Wuxi People’s Hospital after written informed consent was obtained. We selected samples from 5 patients with RAS for whom an irreversible decrease in the FEV1 to <80% of baseline and an irreversible decrease in the TLC to < 90% of baseline were detected. All of these patients with RAS presented with characteristics of interstitial lung disease on radiological examination. Patients whose number of posttransplant days was similar to that of patients with RAS were subsequently selected. The detailed information of the plasma samples is displayed in the Method S1. The baseline information of the patients is displayed in Table 2.

### Histopathological evaluation of the established CR rat model

The same anatomical region of the lung graft was fixed in 10% formalin for 72 hours, embedded in paraffin, and sectioned. HE staining, MT staining, and Sirius red staining were carried out according to standard protocols. The A and B grades were evaluated using standard HE staining on the basis of the International Society for Heart and Lung Transplantation (ISHLT) criteria^41^. Quantitative assessments of endothelialitis, vascular fibrosis, P-AF, O-AF, epithelial hyperplasia, and epithelial flattening were performed on the basis of a previous study by Martinu et al.^42^. The counts of obliterative and non-obliterative bronchioles were determined in the whole field of the section, and the percentage of OB in each lung sample was calculated. The percentage of parenchymal fibrosis was quantified using ImageJ in five random MT-stained fields (20× objective magnification). The pleural thickness was measured in five randomly selected fields (20× objective magnification) of HE-stained specimens, and eight random regions were measured in each visual field of the pleura. All semiquantitative results were determined by two experienced investigators in a blinded manner.

### Exosome extraction and purification

Snap‑frozen tissues were cryosectioned, dissociated in cell dissociation buffer at 37 °C for 15 min to obtain a single‑cell suspension, filtered through 70 μm strainers, and supplemented with protease and phosphatase inhibitors (1:100). The fluids were centrifuged for 10 min at 300 × *g* at 4 °C, 10 min at 2000 × *g* at 4 °C, and 20 min at 10000 × *g* at 4 °C. The supernatant was filtered through a 0.22 μm filter membrane, and then centrifuged for 120 min at 150,000× *g* at 4 °C. The supernatant was subsequently discarded, and the exosomes in the precipitate were resuspended in cold PBS supplemented with protease inhibitors and phosphatase inhibitors. Finally, a size exclusion column was used to purify the exosomes. Four to seven fractions were gathered and added to a 100-kDa Amicon Ultra‒15 ultrafiltration tube (Millipore, UFC910024) for concentration. The particle concentration and size distribution of the exosomes from each sample were estimated using a nanoFCM flow nanoAnalyzer (NanoFCM Co.).

### Western blotting

Exosome protein concentrations were determined using a Bicinchoninic Acid Protein Assay Kit (Thermo). Exosomes (10 µg of protein) were added to loading buffer, and the western blotting was performed as previously reported^43^. The primary antibodies used were as follows: anti-Tsg101, ab125011, Abcam; anti-Alix, ab186429, Abcam; anti-HSP70, ab181606, Abcam; and anti-Calnexin, 10427‒2‒AP, Proteintech.

### ELISA

Sandwich ELISA was performed on 96-well ELISA plates using human ILK (ZC‒58459), MRC1 (ZC‒54588), and NT5E (ZC‒31822) ELISA kits (ZCIBIO, Shanghai, China). All the experimental steps were performed in strict accordance with the instructions.

### Transmission electron microscopy

Exosomes (10 µL) were deposited onto a copper grid, incubated for 10 min at room temperature, washed with distilled water, and blotted dry. The grid was then negatively stained with 10 µL of 2% uranyl acetate for 1 min, dried under an incandescent lamp for 2 min, and examined under a transmission electron microscope at 80 kV for morphology observation.

### PBA-based single-vesicle membrane proteomics

Because the pathological features of the Syn group were consistent across individual animals, a randomly selected subset of three Syn rats was used for sequencing of graft‑derived exosomes together with all CR samples (total of 12 samples) to minimize batch effects. The surface proteins on individual exosomes were profiled using PBA (ExoSeek® panel260, Secretech, Shenzhen, China), which leverages a panel of 260 unique oligonucleotide-labeled antibodies to detect 260 proteins in the membranes of exosomes simultaneously^19^. The libraries were subjected to PE150 sequencing with the DNBSEQ‒T7 platform (MGI, Shenzhen, China), and subsequently analyzed with the software EVisualizer® decoding package (version 1.0, Secretech, Shenzhen, China) to generate an EV ID‒protein expression dataset as the raw PBA data. The membrane protein expression, combination, and exosome subpopulations were then analyzed using EVisualizer®, the detailed methods were displayed in Method S2. Utilizing the CellMarker and Protein Atlas databases, an analysis of the cell source of the exosomes was conducted on the basis of the top 1 membrane protein and the related proteins of the top 1 protein in the PBA and bulk-proteomic data (Data S1).

### 4D-Data-independent acquisition (DIA) proteomic analysis of derived lung grafts

The lung tissues were suspended in protein lysis buffer supplemented with protease inhibitor, and the mixture was analyzed by DIA proteomic analysis provided by Majorbio Company (Majorbio, Shanghai, China). The detailed methods of detection and bioinformatic analysis are described in Method S3.

### Liquid chromatography (LC)- mass spectrometry (MS) based untargeted metabolomic analysis of derived lung grafts

A total of 50 mg of the same anatomical region of lung grafts was submitted to Majorbio Company (Majorbio, Shanghai, China) for untargeted metabolomic analysis. Detailed methods of detection and bioinformatic analysis are described in the Method S4.

### Multiomics WGCNA

To perform WGCNA, we used the R WGCNA package (https://cran.r-project.org/package=WGCNA). For the proteomic WGCNA, the parameters were as follows: power = 4, minimum module size = 30, and minimum height for merged modules = 0.141. For the metabolomic WGCNA, the parameters were as follows: power = 7, minimum module size = 30, and minimum height for merged modules = 0.10785.

### Multiomics correlation analysis

The multiomic correlations between two variables, including the percentages of the 14 exosome clusters, protein expression, metabolite expression, immunocyte proportions, and pathological grade, were analyzed. The mean absolute correlation coefficient (|r|) values for the exosome–protein–pathological feature and the exosome–metabolite–pathological feature relationships were subsequently assessed.

### Statistical analysis

Comparisons of continuous variables were performed using Student’s t-test, whereas the Mann–Whitney U test was used to compare the rank variables between groups. Correlations between continuous variables were evaluated by Pearson correlation tests, whereas the noncontinuous variables were evaluated by Spearman correlation tests. Statistical analyses were performed with GraphPad Prism Software. *P* values < 0.05 were considered to indicate statistical significance.

## Supplementary information

Supplementary Material

Supplementary Data for Cell Source Analysis

Supplementary Data for PBA Sequence

## Data Availability

All of the data are accessible within the main text or supplemental materials. The analytical data used to determine the cellular origins of each exosome subpopulation are displayed in Data S1. The raw protein expression data matrices for PBA‑based single‑vesicle proteomic analysis are displayed in the Data S2. The raw data pertaining to non-targeted metabolomics is available in the MetaboLights database [MTBLS14797; the review link before publication: https://www.ebi.ac.uk/metabolights/reviewerbe7a3c6a-4070-404b-947f-4e7520501ce4], while the raw data for graft protomics can be accessed through the iProX database [IPX0016706000, the review link before publication: https://www.iprox.cn/page/PSV023.html;?url=1782612470207Nc86, password: 7VXT].
