# Supplementary material for: An integrated multiomic single-vesicle atlas delineates graft exosome heterogeneity in chronic lung allograft dysfunction
Source: Signal Transduct Target Ther. 2026 Sep 17;11:389. doi: 10.1038/s41392-026-02927-7 (PMC13588815; doi:10.1038/s41392-026-02927-7)
Supplement: Supplementary file 1 [file 41392_2026_2927_MOESM1_ESM.docx]

Supplementary Materials for

An integrated multiomic single-vesicle atlas delineates graft exosome heterogeneity in chronic lung allograft dysfunction

PDong Tian, Xiangyun Zheng , Zengwei Yu, Dapeng Wang, Weiyang Chen, Yaling Liu, Zehui Liu, Boyang Xia, Xiaohan Jin, Haoji Yan, Ye Wu, Yongsheng Zhao, Chuanfen Zhang, Qiang Pu, Jingyu Chen, Masaaki Sato, Lunxu Liu

Correspondence to: 22tiandong@wchsu.cn; satom-sur@h.u-tokyo.ac.jp; lunxu_liu@aliyun.com.

**This PDF file includes:**

Materials and Methods

Figures. S1 to S27

Tables S1

Captions for Data S1 to S2

**Other Supplementary Materials for this manuscript include the following:**

Data S1 to S2 [Supplemental data 1; Supplemental data 2-PBA data]

Materials and Methods

Method S1

Blood samples were collected from patients using ethylenediaminetetraacetic acid anticoagulation tubes. The collected blood was centrifuged at 1500 × g for 20 min at 4°C to remove blood cells. The supernatant was obtained and centrifuged at 3000 × g for 15 min at 4°C, after which the supernatant was collected. The samples were stored at -80°C.

Method S2

The total expression of each protein was summarized and subsequently normalized using the trimmed mean of M‒values algorithm. The coexpression of two or more proteins on the same single exosome was summarized and subsequently normalized to the count per million. For differential analysis, normality and homogeneity were first examined through the Shapiro‒Wilk test and F test or Bartlett’s test. Student’s t‒test was used to compare the data of two groups if the data were normally distributed and had homogeneous variance. The Mann‒Whitney U test was used if the data were not normally distributed. The Welch’s t‒test was used for data with a normal distribution but not homogeneous variance. The Benjamini‒Hochberg (BH) method was used to adjust the P values. To generate exosome subpopulations, the unsupervised FlowSOM algorithm was used and the distributed stochastic neighbor embedding (t‒SNE) method was employed for plotting.

Method S3

The lung tissues were suspended in protein lysis buffer supplemented with protease inhibitor, and the mixture were treated 3 times in a high-flux tissue grinding machine for 40 s each time. The mixture was then incubated on ice for 30 min and then vortexed for 5–10 s every 5 min. After centrifugation at 16000 × g at 4°C for 30 min, the concentration of protein in the supernatant was measured by a BCA protein assay kit (Thermo Scientific). For further quality control, SDS‒PAGE electrophoresis was performed. Afterwards, a total of 100 μg of protein was resuspended in triethylammonium bicarbonate buffer (final concentration, 100 mM). The mixture was reduced with tris(2-carboxyethyl)phosphine (final concentration, 10 mM) at 37°C for 60 min and alkylated with iodoacetamide (final concentration, 40 mM) at room temperature for 40 min in the dark. Following centrifugation for 20 min at 10000 × g at 4°C, the pellet was resuspended in 100 μL triethylammonium bicarbonate buffer for a final concentration of 100 mM. After incubation at 37°C overnight with trypsin at a 1:50 trypsin-to-protein mass ratio, the liquid portion of the peptide solution were evaporated by a vacuum pump. Afterwards, 0.1% trifluoroacetic acid was added to the enzymatically extracted peptides, after which they were desalted using HLB, and the liquid portion of the peptide solution was evaporated by a vacuum concentrator. Finally, the peptides were quantified using the Peptide Quantification Kit (Thermo Fisher Scientific, 23275). Next, the peptides were analyzed by a Vanquish Neo system coupled with an Orbitrap Astral mass spectrometer (Thermo, USA) at Majorbio Bio-Pharm Technology Co., Ltd. (Shanghai, China). Briefly, an ES906 column (Thermo, USA) was used with solvent A (water with 2% ACN and 0.1% formic acid) and solvent B (water with 80% ACN and 0.1% formic acid). The peptides were eluted using a 60 SPD gradient at a flow rate of 500 nL/min. DIA data were acquired using an Orbitrap Astral mass spectrometer operated in DIA mode. MS data were collected from 100–1700 m/z.

Spectronaut software (version 18) was used to search the raw DIA data. For quantitative analysis, six peptides per protein and three daughter ions per peptide were selected (protein FDR ≤ 0.01, peptide FDR ≤ 0.01, peptide confidence ≥ 99%, and XIC width ≤ 75 ppm). The shared peptides and modified peptides were excluded, and the peak areas were calculated and summed to obtain the quantitative results. Only the proteins that had at least one unique peptide were used for protein identification. Bioinformatic analysis of the proteomic data was performed with the Majorbio Cloud platform (https://cloud.majorbio.com). P values and fold changes (FCs) for the proteins between the two groups were calculated using the R package “t test”. The thresholds of FC (>1.2 or <0.83) and P value < 0.05 were used to identify differentially expressed proteins. The functional annotation of all the identified proteins was performed using GO (http://geneontology.org/) and KEGG pathway analysis (http://www.genome.jp/kegg/). DEPs were further used for GO and KEGG enrichment analysis. Protein‒protein interaction analysis was performed using String v11.5 and Cytoscape software.

Method S4

A total of 50 mg from the same anatomical region of the lung graft was added to a 2 mL centrifuge tube, and 6 mm diameter grinding beads were added. Metabolites were extracted using 400 μL of extraction solution (methanol:water = 4:1 (v:v)) containing 0.02 mg/mL of an internal standard (L-2-chlorophenylalanine). A frozen tissue grinder was used to grind the samples for 6 min (-10°C, 50 Hz), followed by ultrasonic extraction for 30 min (5°C, 40 kHz). After being frozen at -20°C for half an hour, the samples were centrifuged at 13000 × g for 15 min at 4°C, and the resulting liquid was transferred to the injection vials for LC‒MS/MS examination. All the samples were mixed in equal volumes to prepare pooled quality control samples, which were injected every 5 samples to monitor the stability of the analysis. LC‒MS/MS analysis was conducted on a ABSCIEX UHPLC-Triple TOF system equipped with an ACQUITY HSS T3 column (100 mm × 2.1 mm i.d., 1.8 μm; Waters, USA) at Majorbio Bio-Pharm Technology Co., Ltd. (Shanghai, China). The mobile phases consisted of 0.1% formic acid in water:acetonitrile (95:5, v/v) (solvent A) and 0.1% formic acid in acetonitrile:isopropanol:water (47.5:47.5:5, v/v/v) (solvent B). The flow rate was 0.40 mL/min, and the column temperature was 45°C. Mass spectrometry analysis was conducted using a ABSCIEX UHPLC-Triple TOF system. Data acquisition utilized the data-dependent acquisition mode, with detection spanning a mass range of 50–1200 m/z. Quality control (QC) samples are prepared by pooling equal volumes of the extracts from all study samples. Each QC sample had the same volume as the test samples and was processed and detected using the same methods as the analytical samples. During instrumental analysis, a QC sample was inserted after every 5 to 15 analytical samples to monitor the stability of the overall analytical process. The raw data were processed in Progenesis QI for peak detection and alignment, which resulted in m/z values and peak intensity information. Accurate masses were used to identify the mass spectra of metabolic features.

The pretreatment of the LC/MS raw data was performed with Progenesis QI (Waters Corporation, Milford, USA) software. The metabolites were identified by the HMDB (http://www.hmdb.ca/), Metlin (https://metlin.scripps.edu/) and Majorbio databases. The data matrix obtained by searching the databases was uploaded to the Majorbio cloud platform (https://cloud.majorbio.com) for data analysis. At least 80% of the metabolic features detected in any set of samples were retained. After filtering, for specific samples whose metabolite levels were below the lower limit of quantification, the minimum metabolite value was estimated, and each metabolic signature was normalized to the sum. The response intensities of the sample mass spectrometry peaks were normalized using the sum normalization method. Moreover, the variables of the QC samples whose relative standard deviation was > 30% were excluded, and converted to log10 to obtain the final data matrix for subsequent analysis. Afterwards, the R package “ropls” (version 1.6.2) was used to perform principal component analysis (PCA), orthogonal least partial squares discriminant analysis (OPLS-DA), and 7-cycle interactive validation to evaluate the stability of the model. Variable significance in the projection (VIP) was calculated by the OPLS-DA model, and p values generated by Student's t tests were used to identify significantly different metabolites (VIP>1, p<0.05). Differentially abundant metabolites among the two groups were mapped to their biochemical pathways through metabolic enrichment and pathway analysis on the basis of the KEGG database (http://www. genome.jp/kegg/) using the Python package “scipy.stats” (https://docs.scipy.org/doc/scipy/).

Figure. S1.

**Size distribution profiles of exosomes in individual samples across the two groups, acquired via nanoflow technology.** The y‑axis denotes exosome concentration (particles/mL), and the x‑axis indicates particle size (nm).

Figure. S2.

**Full-length Western blots probed for TSG101, Alix, HSP70, and calnexin in exosomes.** The leftmost lane (lane 1) shows the protein molecular weight marker, while lanes 2 and 3 correspond to the control lysate (CL) and the Syn/CR groups, respectively.

Figure. S3.

**The expression of 260 proteins on the surface of exosomes.**(a) Principal component analysis (PCA) and multidimensional scaling (MDS) plots (left and right, respectively) reveal the global differences in single-vesicle proteomic profiles among samples. (b-c) A dot plot (b) and a corresponding heatmap (c) are shown to illustrate the abundance of these 260 membrane proteins on exosomes.

Figure. S4.

**Protein-protein interaction (PPI) network of differentially expressed proteins between the Syn and CR groups.** Colored nodes denote proteins, and edges represent interactions. Line thickness reflects association strength.

Figure. S5.

**KEGG enrichment analysis of differentially expressed proteins associated with exosome clusters 1–6.**

Figure. S6.

**KEGG enrichment analysis of differentially expressed proteins associated with exosome clusters 7–12.**

Figure. S7.

**KEGG enrichment analysis of differentially expressed proteins associated with exosome clusters 13–14.**

Figure. S8.

**Gene Set Enrichment Analysis (GSEA) of differentially expressed proteins.** Circular bar plot presenting the enrichment results, ordered from top to bottom by Gene Ontology (GO) and KEGG categories. The stacked rectangles beneath each bar correspond to 14 exosome subgroups, where colored segments denote subgroups whose proteins are co-enriched in the common pathways.

Figure. S9.

**Stratification of samples by Fn1 (top) and Cd9 (bottom) expression levels (high vs. low).** Heatmaps show the expression profiles of individual samples, and the adjacent bubble plot (right) displays KEGG pathway enrichment among differentially expressed proteins between the two expression groups.e analysis between high vs. Low level expression of Fn1 (top) and Cd9 (bottom) group. Expression heatmaps of individual samples stratified by Fn1 (top) and Cd9 (bottom) expression levels (high vs. low). The bubble plot on the right shows KEGG pathway enrichment among differentially expressed proteins between the high- and low-expression groups.

Figure. S10.

**Contribution of exosome subpopulations to the immune cell landscape during CLAD.** (a) Bar plot comparing the relative abundances of infiltrating immunocytes in grafts from the Syn and CR groups. The cell types assessed comprise naïve B cells, memory B cells, plasma cells, CD8⁺ T cells, CD4⁺ T‑cell subsets (naïve, resting memory, activated memory, follicular helper, and regulatory), γδ T cells, resting and activated NK cells, monocytes, macrophage subtypes (M0, M1, M2), resting and activated dendritic cells, resting and activated mast cells, eosinophils, and neutrophils. (b–c) Heatmaps showing correlations between immune cell frequencies and the abundances of exosome clusters (b) or exosome subgroups of distinct cellular origins (c). The color intensity reflects the correlation coefficient (*r*, from –1 [blue] to 1 [red]), and significance levels are labeled with white text or indicated by asterisks (P < 0.05; *P < 0.01; **P < 0.001; ***P < 0.0001).

Figure. S11.

Heatmap illustrating the expression levels of exosomal membrane protein combinations detected by PBA, categorized into single‑protein combinations (top) and two‑protein pairings (bottom).

Figure. S12.

Heatmap illustrating the expression levels of exosomal membrane protein combinations detected by PBA, categorized into three‑protein (top) and ≥4‑protein (bottom) groups.

Figure. S13.

**Exosome subgroups derived from T cells, CD4⁺ T cells, and CD8⁺ T cells.** The circular heatmap illustrates the abundance of differentially expressed exosomes in each sample, with sample labels positioned at the origin of the circular layout. The color gradient (blue to red, –1 to 1) represents the expression levels of distinct membrane protein combinations. Significance: P < 0.05; *P < 0.01; **P < 0.001; ***P < 0.0001.

Figure. S14.

**Exosome subgroups derived from B cells and macrophages.** The circular heatmap illustrates the abundance of differentially expressed exosomes in each sample, with sample labels positioned at the origin of the circular layout. The color gradient (blue to red, –1 to 1) represents the expression levels of distinct membrane protein combinations. Significance: P < 0.05; *P < 0.01; **P < 0.001; ***P < 0.0001.

Figure. S15.

**Exosome subgroups derived from neutrophils.** The circular heatmap illustrates the abundance of differentially expressed exosomes in each sample, with sample labels positioned at the origin of the circular layout. The color gradient (blue to red, –1 to 1) represents the expression levels of distinct membrane protein combinations. Significance: P < 0.05; *P < 0.01; **P < 0.001; ***P < 0.0001.

Figure. S16.

**Annotation of differentially abundant metabolites and the pathway analysis.** (a–b) The bar plots show the annotated secondary (a) and detailed (b) pathways of the differentially abundant metabolites. (c) The iPath plot illustrates the differentially abundant metabolites and proteins in the metabolism network.

Figure. S17.

**The commonly enriched pathways of differentially expressed proteins and differentially abundant metabolites.** The bar plots show the commonly enriched pathways of differentially expressed proteins (blue) and differentially abundant metabolites (red).

Figure. S18.

**Correlation analysis between the proportions of immunocytes and metabolite expression.** Correlation heatmap between the proportions of immunocytes and metabolite expression. The depth of the color represents the r value (blue to red, -1 to 1).

Figure. S19.

**KEGG enrichment analysis of the differentially expressed proteins related to the cluster 9–14 exosomes.** (a–f) The bar plots illustrate the enriched pathways of cluster 9 (a), cluster 10 (b), cluster 11 (c), cluster 12 (d), cluster 13 (e), and cluster 14 (f).

Figure. S20.

**Association between cluster 12 exosomes and glycerophospholipid metabolism.** The KEGG pathway plot displays differentially abundant metabolites and proteins, with metabolites belonging to cluster 12 circled. Red indicates upregulation and blue indicates downregulation in the CR group relative to the control.

Figure. S21.

**Intersection analysis of differentially expressed proteins and differentially abundant metabolites.** Venn diagrams showing the intersection analysis of differentially expressed proteins and differentially abundant metabolites associated with clusters 1, 2, and 4 (top) and clusters 5, 12, and 13 (bottom).

Figure. S22.

**WGCNA of differentially expressed proteins.** The dendrogram shows protein clustering based on their associations with pathological and immune features. Each leaf on the tree represents a protein, and the heatmap cells are color‑coded according to the correlation coefficient between each feature and protein expression, with red indicating high correlation.

Figure. S23.

**WGCNA of differentially expressed proteins.** The plot shows the module traits related to pathological and immune features. The different modules of the trunk branch are marked with different colors. The module name is displayed on the left, with each row corresponding to a module and each column corresponding to a feature. Each cell uses a different color to represent the correlation coefficient between the module and the features, and a high correlation indicated in dark red or blue.

Figure. S24.

**WGCNA of differentially expressed metabolites.** The dendrogram shows protein clustering based on their associations with pathological and immune features. Each leaf on the tree represents a metabolite, and the heatmap cells are color‑coded according to the correlation coefficient between each feature and metabolite expression, with red indicating high correlation.

Figure. S25.

**WGCNA of differentially expressed metabolites.** The plot shows the module traits related to pathological and immune features. The different modules of the trunk branch are marked with different colors. The module name is displayed on the left, with each row corresponding to a module and each column corresponding to a feature. Each cell uses a different color to represent the correlation coefficient between the module and the features, and a high correlation indicated in dark red or blue.

Figure. S26.

**The size and concentration of the exosomes were assessed by nanoflow technology.** For each sample, the left bar plots show the distribution of exosome sizes, and the right scatter plot shows the concentration information.

Figure. S27.

**Multiomics network chart.** The charts show how cluster 12 affects CLAD at the protein, metabolite, and immunocyte levels. The green circles indicate exosomes, the red circles indicate pathological features, the blue circles indicate proteins, the purple circles indicate metabolites, and the yellow circles indicate immunocytes. The red lines indicate positive correlations, and the blue lines indicate negative correlations. Line thickness represents the |r| value.

**Table S1.**

Exosome size and concentration.

| **Sample name** | **Exosome size**  **(nm)** | **Exosome concentration**  **(Particles / g tissues)** |
| --- | --- | --- |
| **Syn** | **78.02 ± 0.89** | **3.73 ×10^11^** |
| Syn1 | 78.75 ± 15.67 | 3.86 ×10^11^ |
| Syn2 | 78.27 ± 15.61 | 4.34 ×10^11^ |
| Syn3 | 77.03 ± 15.54 | 3.01 ×10^11^ |
| **CR** | **79.81 ± 3.42** | **8.67 ×10^10^** |
| CR1 | 80.00 ± 20.10 | 6.21 ×10^10^ |
| CR2 | 79.03 ± 20.21 | 5.22 ×10^10^ |
| CR3 | 81.45 ± 21.43 | 8.35 ×10^10^ |
| CR4 | 83.96 ± 22.79 | 4.24 ×10^10^ |
| CR5 | 78.96 ± 16.45 | 2.17 ×10^11^ |
| CR6 | 82.26 ± 18.60 | 1.06 ×10^11^ |
| CR7 | 80.73 ± 18.57 | 5.64 ×10^10^ |
| CR8 | 71.73 ± 14.12 | 7.82 ×10^10^ |
| CR9 | 80.19 ± 16.68 | 8.25 ×10^10^ |
| ***P*** | 0.4030 | **< 0.0001** |

Data S1. (separate file)

Data for cell source analysis. This supplementary dataset contains the analytical data used to determine the cellular origins of each exosome subpopulation, including the provenance data of the top marker proteins for each subpopulation.

Data S2. (separate file)

Original data of PBA-based single-vesicle proteomics. This supplementary dataset contains the raw protein expression data matrices for each sample from the PBA‑based single‑vesicle proteomic analysis.
